# Surface modification of XSe (X = Cu and Ag) monolayers by grope 1 elements: A metal to semiconductor transition by a first-principles perspective

**DOI:** 10.1038/s41598-024-63580-0

**Published:** 2024-06-03

**Authors:** A. Bafekry, M. Faraji, S. Hasan Khan, M. M. Fadlallah, H. R. Jappor, B. Shokri, M. Ghergherehchi, Gap Soo Chang

**Affiliations:** 1 Department of Physics, University of Guilan, Rasht, 41335-1914 Iran; 2 Department of Physics, Shahid Beheshti University, Tehran, 19839-63113 Iran; 3 Micro and Nanotechnology Graduate Program, TOBB University of Economics and Technology, Sogutozu Caddesi No 43 Sogutozu, 06560 Ankara, Turkey; 4 Department of Electrical and Electronic Engineering (EEE), Khulna University of Engineering and Technology (KUET), Khulna, 9203 Bangladesh; 5 Department of Physics, Faculty of Science, Benha University, Benha, 13518 Egypt; 6 Department of Physics, College of Education for Pure Sciences, University of Babylon, Hilla, Iraq; 7 Physics Department and Laser-Plasma Research Institute, Shahid Beheshti University, Evin, Tehran, 19839- 69411 Iran; 8 College of Electronic and Electrical Engineering, Sungkyunkwan University, Suwon, Korea; 9 Department of Physics and Engineering Physics, University of Saskatchewan, Saskatoon, SK S7N5E2 Canada

**Keywords:** Materials science, Physics

## Abstract

Two-dimensional (2D) materials can be effectively functionalized by chemically modified using doping. Very recently, a flat AgSe monolayer was successfully prepared through direct selenization of the Ag(111) surface. Besides, the results indicate that the AgSe monolayer like CuSe, has a honeycomb lattice. Motivated by the experimental outcomes, in this work, employing first-principles calculations, we systematically investigate the electronic and optical properties of AgSe and CuSe monolayers, as well as the impact of alkali metals (Li, Na and K). Without functionalization, both the CuSe and AgSe monolayers exhibit metallic characteristics. The Li (Na)-CuSe and Na (K)-AgSe systems are dynamically stable while, the K- and Li-CuSe and Li-AgSe are dynamically unstable. Interestingly, the functionalized CuSe system with Li and Na atom as well as AgSe with K and Na atom, can open the band gaps, leading to the actualization of metal to semiconductor transitions. Our results show that, the electronic characteristics of the Na-CuSe/AgSe system can be modulated by adjusting the adsorption heights, which gives rise to the change in the electronic properties and the band gap may be controlled. Furthermore, from the optical properties we can find that the K-AgSe system is the best candidate monolayer to absorb infrared radiation and visible light. Consequently, our findings shed light on the functionalization of 2D materials based CuSe and AgSe monolayers and can potentially enhance and motivate studies in producing these monolayers for current nanodevices and future applications.

## Introduction

Since the remarkable experimental fabrication of graphene^1^, a fast-developing family of 2D materials has emerged, including phosphorene^2^, silicene^3^, antimonene^4^, hexagonal boron nitrides (h-BN)^5,6^, arsenene^7,8^, germanene^9^, MXenes^10,11^, Janus monolayers^12–15^ and transition metal dichalcogenides (TMDs)^16–23^ have aroused worldwide research attention due to their intriguing and unique features, as well as their potential promising applications in a variety of fields such as gas sensor, optoelectronic devices, photocatalysts, spintronic, and thermoelectric applications and so on^24–29^. Researchers have shed light on the 2D transition metal monochalcogenides (TMM) family in the last few years. Studies of these 2D materials have not only expanded the 2D materials collections but also provided many exotic physical and chemical properties and possible applications in high-speed nano-devices^30–33^. For example, it has been established that the synthesized CuAs monolayer has a potentially adjustable bandgap^34^. In addition, it has been demonstrated that monolayer AgTe, which was experimentally released by Liu et al. Liu et al.^30^ possess electronic features of the Dirac nodal line fermions (DNLFs). Besides, spin-orbit coupling (SOC) is strongly connected to the stability of these DNLFs, which typically necessitates an additional crystalline symmetry to safeguard the degeneracy in actual materials^35,36^.

Recent efforts have been exploited to construct 2D TMM, including selenium-based TMM, like copper selenide (CuSe) and silver selenides (AgSe). First, the novel monolayer TMM CuSe was characterized and synthesized by molecular beam epitaxy on Cu(111) surface with and without triangular nanopores^31^. Very recently, Wang et al. experimentally demonstrated the presence of triangular nanoholes and the honeycomb network of the CuSe monolayer^37^. However, up to now, two types of atomic configurations have been found in the fabricated CuSe monolayer: the planar and low-buckled phases. The phonon dispersion curves of these phases show that planar honeycomb CuSe is more stable than the low-buckled phase, which is unstable due to the presence of imaginary frequencies^38^. It is worth noting that the planar CuSe has a hexagonal lattice with a periodicity of about 3 nm and owns two 2D DNLFs safeguarded by mirror reflection symmetry^31^. Moreover, the CuSe monolayer exhibits negative differential resistance and powerful electrical anisotropy^39^. As a result, CuSe has a high opportunity to form fascinating 2D DNLFs and offers a wide range of possible applications in photoelectronic nanodevices and electrical anisotropy-based.

Meanwhile, bulk silver selenides are among the TMMs that have grabbed widespread attention due to their outstanding transport, thermoelectric, electrical, and optical properties^40–42^. Most importantly, even if obtaining silver selenide monolayer with varying stoichiometric ratios is a significant difficulty, very recently, Lu et al. successfully prepared a flat AgSe monolayer through direct selenization of the Ag(111) surface^43^. It was demonstrated that the AgSe monolayer is stable in ambient conditions at room temperature. Besides, the results have shown that the AgSe monolayer is like CuSe, has a honeycomb lattice, and possesses two 2D DNLFs safeguarded by mirror reflection symmetry without considering the SOC, which makes it a perfect option for producing interesting 2D DNLFs. However, CuSe and AgSe monolayers are undesirable for prospective use within electronic components due to their metallic character. The materials used in optoelectronics require a semiconducting property. The researchers detected a bandgap of up to 1 eV and Dirac points close to the Fermi level. Thus, appropriate doping of these monolayers, or opening an energy gap, can turn them into semiconductors; this technique has previously successfully modified the electronic properties of many graphene-like 2D materials^44–53^.

Motivated by the experimental results that fabricated AgSe and CuSe monolayers, we used first-principles calculations to examine the impact of Li, Na, and K functionalization on the structural, optical, and electronic properties of AgSe and CuSe monolayers. Our findings manifested that the stable AgSe and CuSe monolayers are metals, and the functionalization opens bandgaps near the Fermi level, leading to the actualization of metal-to-semiconductor transitions. As a result, our research clarifies the decorative features of these 2DM monolayers and may encourage further research into their production for use in present-day nanodevices and future uses.

## Method

 We use density functional theory as implemented in the Vienna ab-initio simulation package (VASP)^56,57^ to calculate the electronic structure of the nanosheets. The plane-wave basis projector augmented wave (PAW) method was used with the generalized gradient approximation of Perdew-Burke-Ernzerhof (PBE)^54,55^. The Heyd-Scuseria-Ernzerhof (HSE06)^58^ functional employed to obtain more accurate bandgap values. The kinetic energy cut-off was taken to be 500 eV for the plane-wave expansion, and the system’s energy was converged to below 10^−5^ eV. The Hellmann-Feynman forces converged to below 0.05 eV/Å for atomic relaxation. A 21 21 1 Γ-point centered Monkhorst-Pack^59^ *k*-point grid was employed for the unit cells. Charge transfer was calculated using the Bader method^60^. The long-range vdW interactions were considered by including Van der Waals (vdW) corrections^61^. The phonon dispersion curves were obtained using the small displacement technique employed in the PHONOPY code^62^.

## CuSe and AgSe monolayer

**Figure 1:**
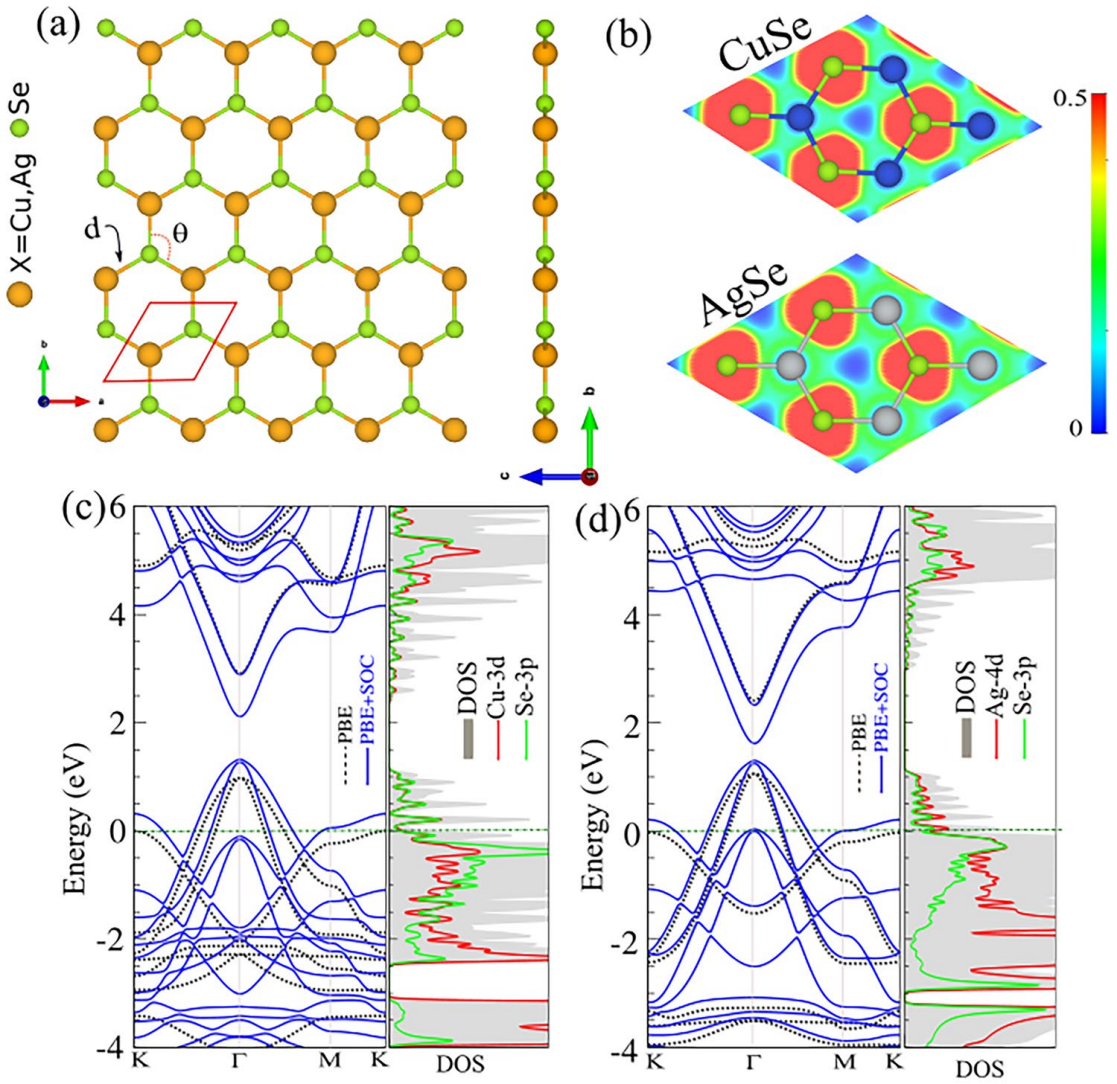
(**a**) Geometric atomic structure (**b**) contour plot of the electron localization function (ELF) and (**c**) electronic band structure with corresponding DOS and PDOS of CuSe/AgSe monolayers.

First, we have examined the pristine CuSe and AgSe monolayers and their structural and electronic properties. The geometric structures of the CuSe and AgSe monolayers with their unit cell (red parallelogram), is shown in the Fig. 1a. We find that, the CuSe and AgSe monolayers, having non-buckling, planar structures, belong to the *P3m1* symmetry group. The calculated lattice constant, *a*, (bond length, *d*) for AgSe is 3.94 Å (2.27 Å) and for CuSe is 4.27 Å (2.47 Å). The bond angles between Ag and Se atom and Cu and Se atom are 119.3*°* and 120.12*°*, respectively. The cohesive energy is calculated as follows, *E*_*coh*__=_ (*E*_*tot*_ − * E*_*X*_ − *E*_*Se*_)/2 which, *E*_*X*_ (X = Cu, Ag) and *E*_*Se*_ represent the energy of the free-standing Cu (Ag) atom and Se atom, respectively. *E*_*tot*_ signifies the total energy of the CuSe (AgSe) monolayer, and two is for the number of atoms of the primitive unit cell. Both monolayers have negative cohesive energy, suggesting the exothermic feasibility of the monolayers. However, the CuSe monolayer has the most negative cohesive energy compared with the AgSe monolayer, also suggesting the superior exothermic feasibility of the CuSe monolayer. The charge transfer of the CuSe and AgSe monolayers is calculated as shown in the Table 1. The calculated values are 0.4 *e* and 0.3 *e* for the CuSe and AgSe monolayers, respectively. These values show, meaning the charge transferring occur from Cu and Ag atom to Se atom. The electron localization contour simulated for the monolayers is also depicted in Fig. 1b. The contour plots depict the electron localization between Cu (Ag) and Se atoms, confirming their strong bonding. The details of structural and electronic parameters are also listed in Table 1. The work function is calculated as: and the calculated Φ of CuSe and AgSe monolayers are 5.73 eV and 5.74 eV, respectively. These values are further confirmed by electrostatic potential curves, as shown in Fig. S1 of the supplementary information (SI).

**Table 1:** Structural and electronic parameters of XSe monolayers.

System	*a*	*d*	*θ*	*Ecoh*	∆*Q*	Φ
	(Å)	(Å)	(°)	(eV/atom)	(e)	(eV)
CuSe	3.94	2.27	119.93	− 3.03	0.40	5.73
AgSe	4.27	2.47	120.12	− 2.38	0.30	5.74

*a*, Lattice constant; *d*, bond length; *θ*, bond angles; *E*_*coh*_, cohesive energy per atom, ∆*Q*, charge transfer; Φ, work function.

The electronic band structure with corresponding density of states (PDOS) and projected DOS (PDOS) with the PBE level for the CuSe and AgSe monolayers, are shown in Fig. 1c and d, respectively. From the band structure of both monolayers, we can see that the energy bands are crossed the Fermi level, result they are metal. In the CuSe (AgSe) monolayer, the energy bands around of Fermi level is predominantly contributed from the Cu-3*d* (Ag-4*d*) orbital as shown in Fig. 1d DOS/PDOS. To gain more insights into orbital contributions for the CuSe and AgSe monolayers, the orbital projections are shown in Fig. S2. In the CuSe monolayer, the contributing orbitals around the Fermi level are *d*_*xz*_, *d*_*yz*_ and *p*_*z*_ from Cu and Se atoms, respectively. For the AgSe, the contribution is from *d*_*xz*_, *d*_*yz*_ (*p*_*z*_) orbitals of Ag (Se) atom.

## Functionalized monolayers

The adsorption is one of the efficient ways for tunable structural, electronic, and magnetic properties of 2DMs. Alkali metal atoms such as Li, Na and K are potential candidates for such functionalization. In the first step, we find the most stable site of single atom (Li, Na, K) of adsorption for the XSe (X = Cu and Ag) monolayer. A schematic view of the favorable adsorption sites of single atom on the XSe monolayer is indicated in Fig. S3a. The most stable site of adsorption is obtained by placing the single atom to four preferable adsorption sites at an initial height of 2 Å from the surface of XSe monolayer. The most state site among the four different sites of atoms have been determined by fully structural optimizations in all directions. Different adsorption sites of Li, Na and K on XSe given with respect to the position; (1) Top-Se (top site above a Se atom ((T_Se_), (2) Top-X (the top site above a X atom (T_X_), (3) Hollow (above the center of a hexagon with six X and Se atoms (H_XSe_), (4)

Bridge (above the middle of a X-Se bond (B_XSe_). For instance, we find the most stable site of Li atom on the CuSe monolayer. The most feasible site is selected from four possible adsorption sites for the Li single atom on the CuSe monolayer surface, according to the relative total energies are shown in Fig. S3b. After structural optimization, the most stable site configuration among the different adsorption sites is determined H_XSe_. Once the favorable adsorption sites of single atoms are examined, the possible functionalization of XSe monolayer is considered in the H_XSe_ configuration. Hereafter, for instance, the CuSe monolayer functionalized with Na atom simply written as Na-CuSe.

## Structural properties

**Figure 2:**
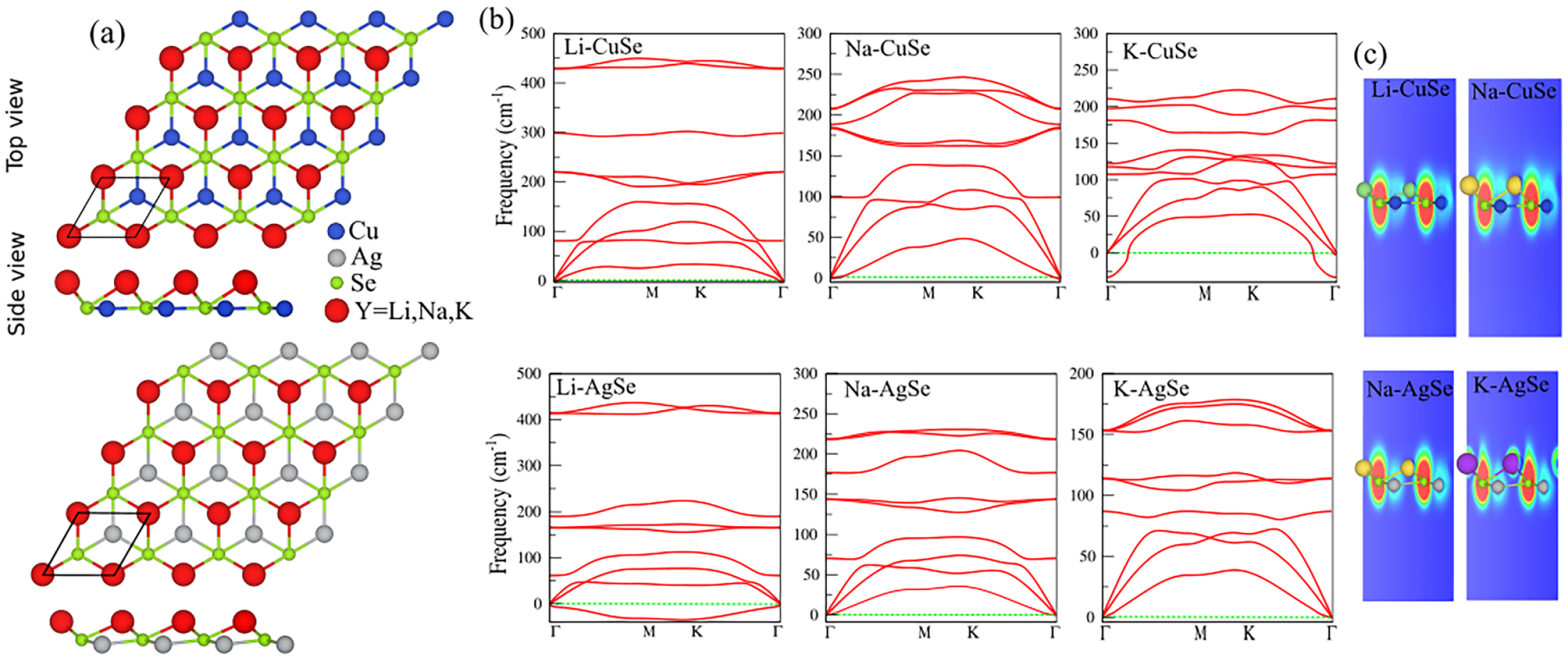
**a** Geometric atomic structure, **b** phonon band dispersion and **c** contour plot of the ELF for the functionalized CuSe (top) and AgSe (bottom) monolayers. The primitive unit cell is indicated by a red parallelogram.

To tune the electronic and optical properties of the CuSe and AgSe monolayers, the alkali metals (Group I) atoms including Li, Na and K atoms are functionalized in these structures. The geometry-relaxed functionalized structures are shown in Fig. 2a. A black parallelogram denotes the unit cell of the functionalized structure and consists of 3 atoms: Cu (Ag), Se, and Group I atom (Y- Li, Na, K). To evaluate the dynamical stability of these structures, phonon dispersion curves are calculated as shown in Fig. 2b. From the structures, the K-CuSe and Li-CuSe systems are found dynamically unstable due to having negative phonon branches in the phonon dispersion curves, as confirmed in Fig. 2b. Though the pristine monolayers are non-buckling, the structures become buckling after atom functionalization. The buckling heights are 1.01, 1.56, 1.47 Å, and 2.03 Å for Li-CuSe, Na-CuSe, Na-AgSe, and K-AgSe systems, respectively. Due to functionalization of the Li, Na, and K atoms, the lattice constants deviate from the pristine monolayers. For the dynamically stable structures they become 4.12 (Li-CuSe), 4.16 (Na-CuSe), 4.56 (Na-AgSe) and 4.62 Å (K-AgSe). The bond angles and lengths are also changed and are described in Table 2. The absorption energy of these systems is calculated as follows: According to *E*_*a*_=*E*_*tot*_ − *E*_*P*_ − *E*_*A*_ formula, the absorption energy is calculated, where *E*_*tot*_, *E*_*P*_ and *E*_*A*_ are total energies of decorated structures, pristine structures, and alkali metal atoms (A = Li, Na, and K), respectively. The absorption energy is calculated − 2.18 eV for Li-CuSe, − 1.8 eV for Na-CuSe, − 2.31eV for Na-AgSe, and − 2.16 for K-AgSe systems. From ELF surfaces, as shown in Fig. 2c, the functionalized atoms are strongly covalently bonded with the monolayer, suggesting structural stability. The charge transfer is also sensitive to atom functionalization, becoming the highest in Li-CuSe system. Moreover, the charge accumulation occurs in all systems while the pristine monolayers deplete the charges.

**Table 2:** Structural and electronic parameters of functionalized CuSe and AgSe monolayers with X (Li, Na, K) atoms, as shown in Fig. 2.

System	*a*	*d*1*,*2	*θ*	*h*	*E*_*a*_	∆*Q*	Φ
	(Å)	(Å)	(°)	(Å)	(eV/atom)	(e)	(eV)
Li-CuSe	4.12	2.58,2.38	105.65	1.01	− 2.18	0.85	4.35
Na-CuSe	4.16	2.86,2.40	93.08	1.56	− 1.80	0.75	4.40
Na-AgSe	4.54	2.87,2.64	104.53	1.47	− 2.31	0.78	4.53
K-AgSe	4.62	3.17,2.69	93.64	2.03	− 2.16− 2.16	0.74	4.65

*a*, Lattice constant; *d*_2_, bond length between X–Se *d*_1_ and Cu/Ag–Se atoms; *θ*, bond angles between Se–X–Se atoms; *h*, height;* E*_*a*_, adsorption energy per atom; ∆*Q*, charge transfer from X atom to neighbor atoms; Φ, Work, function.

## Electronic properties

**Figure 3:**
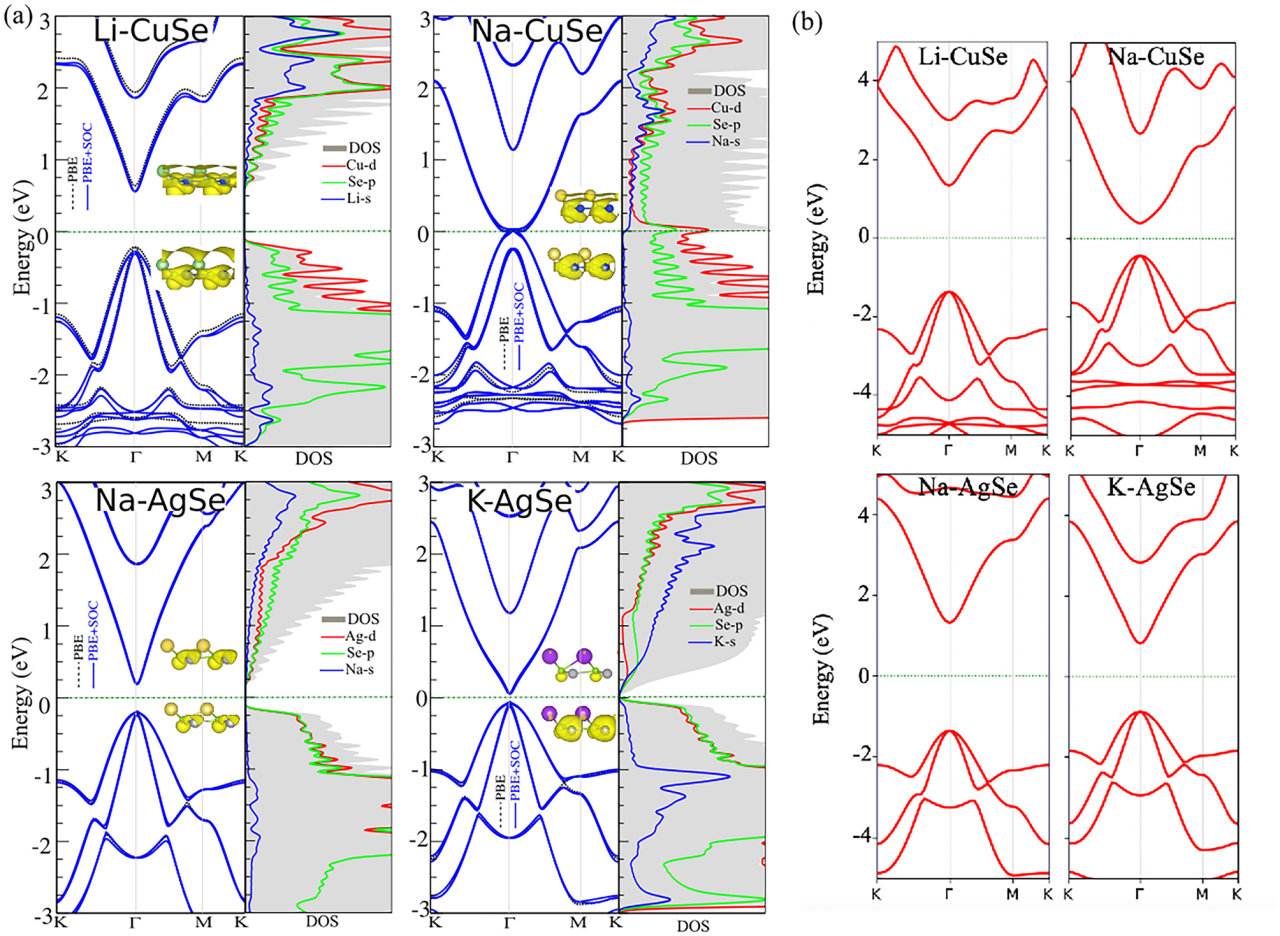
Electronic band structure with corresponding DOS/PDOS with considering (**a**) PBE/PBE + SOC and (**b**) HSE06 levels for the Li-CuSe, Na-CuSe, Na-AgSe and K-AgSe systems. Charge densities of the VBM and CBM orbitals are indicated as insets. The zero energy is set to the Fermi level.

The band structure of the dynamically stable structures is susceptible to atomic functionalization. The band structure with PBE/PBE+SOC with considering DOS/PDOS for the functionalized systems, is shown in Fig. 3a. Interestingly, in the Li-CuSe system, the highest bandgap is opened, suggesting the metal to semiconductor switching in this monolayer. The calculated bandgaps are 0.85 eV (0.81 eV) for Li-CuSe, metal (metal) for Na-CuSe, 0.41 eV (0.38 eV) for Na-AgSe, and 0.13 eV (0.11 eV) for K-AgSe in PBE (PBE+SOC) method. Our results show a metal to semiconductor transition in pristine to functionalized monolayers, while among these structures, Na-CuSe has no bandgap. Notice that, in the Li-CuSe system, the bandgap is direct, and both the VBM and CBM placed at the Γ-point. Considering the SOC effect, the bandgap becomes slightly lower, showing the spin coupling responsiveness of the bandgap. Due to Li-CuSe system, the CBM and VBM are shifted to the lower and upper sides of the Fermi level, causing the bandgap opening. Since these monolayers are semiconductor, the HSE06 functional was also used to study the electronic band structures, shown in Fig. 3b. It is clear that the HSE06 results are consistent with PBE for the type of indirect semiconducting band gap in these functionalized systems. Based on the acquired band structure by HSE06 method, the direct band gap of Li-CuSe, Na-CuSe, Na-AgSe and K-AgSe systems was estimated to be 2.69, 0.82, 2.67 and 1.68 eV, respectively. The electrostatic potential shifting, as shown in Fig. S4, is also one of the responsible factors for the bandgap. As shown in the DOS/PDOS in Fig. 3a, the VBM (CBM) is dominantly contributed by Cu-*d* (Se-*p*) orbitals. The atomic contribution in the VBM and CBM charge density inset, where it is clearly shown that the Li-CuSe structure is assisting the charge density contribution in the VBM and CBM. However, while K-CuSe system, the opposite effect is found, resulting in a metallic state (see Fig. 3b). The DOS/PDOS in Fig. 3b clearly shows that K-CuSe system does not shift the bands, which is also found in the PBE+SOC level. In the Na-AgSe system, again, the bandgap opens at Γ-point in both PBE and PBE+SOC levels, as shown in Fig. 3c. From the DOS/PDOS, the VBM (CBM) is dominated by Se-*p* (Ag-*d*) orbitals and the Na-*s* orbitals contribute to the VBM (CBM) downward (upward) shifting. Again, the Na atom assists the charge density contribution of the Ag and Se atoms in VBM and CBM, as shown in the charge density inset. As shown in Fig.S2(c), the electrostatic potential energy is shifted due to the Na-functionalization, resulting in the bandgap opening.

In the K-AgSe system, the K-*s* orbital is now contributing to CBM while Se-p orbitals for VBM, causing slight bandgap opening in both PBE and PBE+SOC levels as shown in Fig. 3d. The charge density plots also show that K atoms actively contribute to the charge density in CBM. The work function is also highly receptive in atomic functionalization in both monolayers. The work functions become lower due to atomic functionalization, and the lowest work function, 4.35 eV, is found in the Li-CuSe system, suggesting the suitability of photovoltaic application of these monolayers.

**Figure 4:**
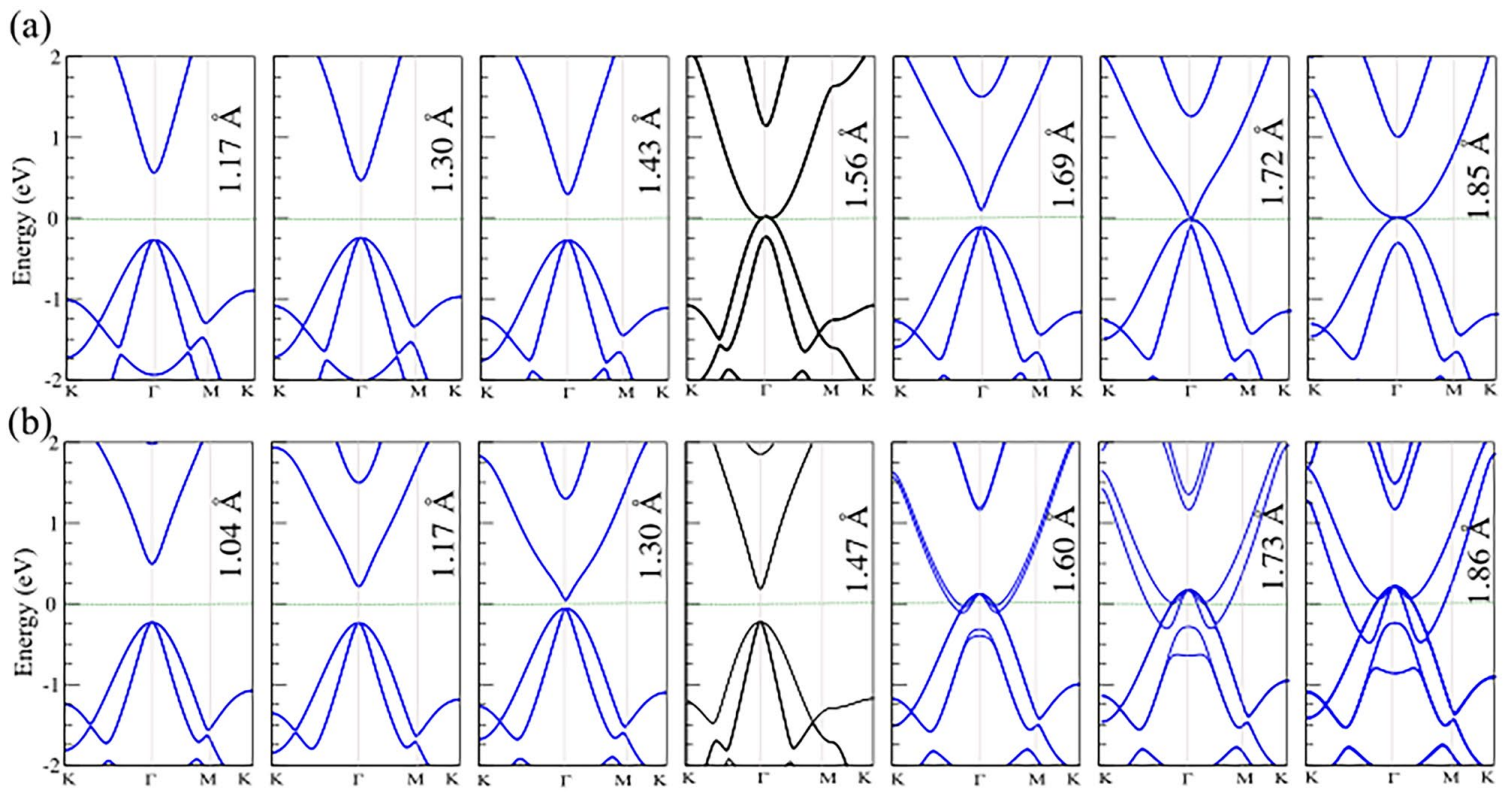
Electronic band structure of functionalized (**a**) Na-CuSe and (**b**) Na-AgSe monolayers for different adsorption heights within PBE level. The zero of energy is set to the Fermi energy.

The electronic band structures of Na-CuSe and Na-AgSe systems for different adsorption heights are depicted in Fig. 4. One can find from the band structures of the Na-CuSe monolayer in Fig. 4a that the decrease in the adsorption heights gives rise to metal-semiconductor transition in the Na-CuSe system. Indeed, when the adsorption height decreases from the equilibrium height of 1.56–1.43 Å, the band gap of Na-CuSe is opened to be 0.55 eV. Further decreasing the adsorption heights to 1.30 Å and 1.17 Å, the band gap of Na-CuSe increases to 0.70 eV and 0.85 eV, respectively. In this case, both the VBM and CBM of Na-CuSe system are located at the Γ point, exhibiting that the Na-CuSe structure possesses a direct band gap semiconductor. This finding suggests that the decrease in the adsorption heights in Na-CuSe not only gives rise to a transition from metal to semiconductor but also increases the band gap. On the other hand, when the adsorption height increases from 1.56 to 1.69 Å, it tends to open a band gap of about 0.22 eV. This finding suggests that the transition from metal to semiconductor appears in the Na-CuSe system, increasing the adsorption height to 1.69 Å. However, when the adsorption height further increases to 1.72 Å and 1.85 Å, such band gap closes, and thus, the Na-CuSe system becomes a metallic system. All discussions above demonstrate that the change in the adsorption heights gives rise to the transition between semiconductor and metal, demonstrating that the Na-CuSe system is a promising candidate for multifunctional nanodevices. As discussed, the Na-AgSe system exhibits a semiconducting characteristic at the equilibrium adsorption height of 1.47 Å. Such semiconducting characteristics can also be modulated by changing the adsorption heights as depicted in Fig. 4b. It can be seen that when the adsorption height increases from 1.47 to 1.60 Å and to 1.86 Å, the band gap of the Na-AgSe monolayer closes, and it becomes a metallic system. On the other hand, when the adsorption height decreases from 1.47 to 1.30 Å, the band gap of the Na-AgSe decreases from 0.41 to 0.15 eV. More interestingly, when the adsorption height continuously decreases to 1.17 Å and 1.04 Å, the band gap of the Na-AgSe starts to rise again to 0.45 eV and 0.75 eV, respectively. Therefore, the change in the adsorption heights in Na-AgSe system gives rise not only to a change in its bandgap but also to the transition from semiconductor to metal.

## Optical properties

Now we discuss the optical properties by calculating the absorption coefficient *α*(*ω*):

1 $$\alpha (\omega )=\sqrt{2}\omega \sqrt{\sqrt{{{\varepsilon }_{Re}^{2}\left(\omega \right)+\varepsilon }_{Im}^{2}(\omega )}-{\varepsilon }_{Re}\left(\omega \right),}$$

as a response under the applied light with angular frequency *ω*. The dielectric function *ε*(*ω*) = *ε*_Re_(*ω*) + *iε*_Im_(*ω*) depends on the electronic structure. The imaginary part (*ε*_Im_) of the complex dielectric function is evaluated using the momentum operator (between the conduction band and valence band), and the real part (*ε*_Re_) is calculated by the Kramers-Kronig relation.

**Figure 5:**
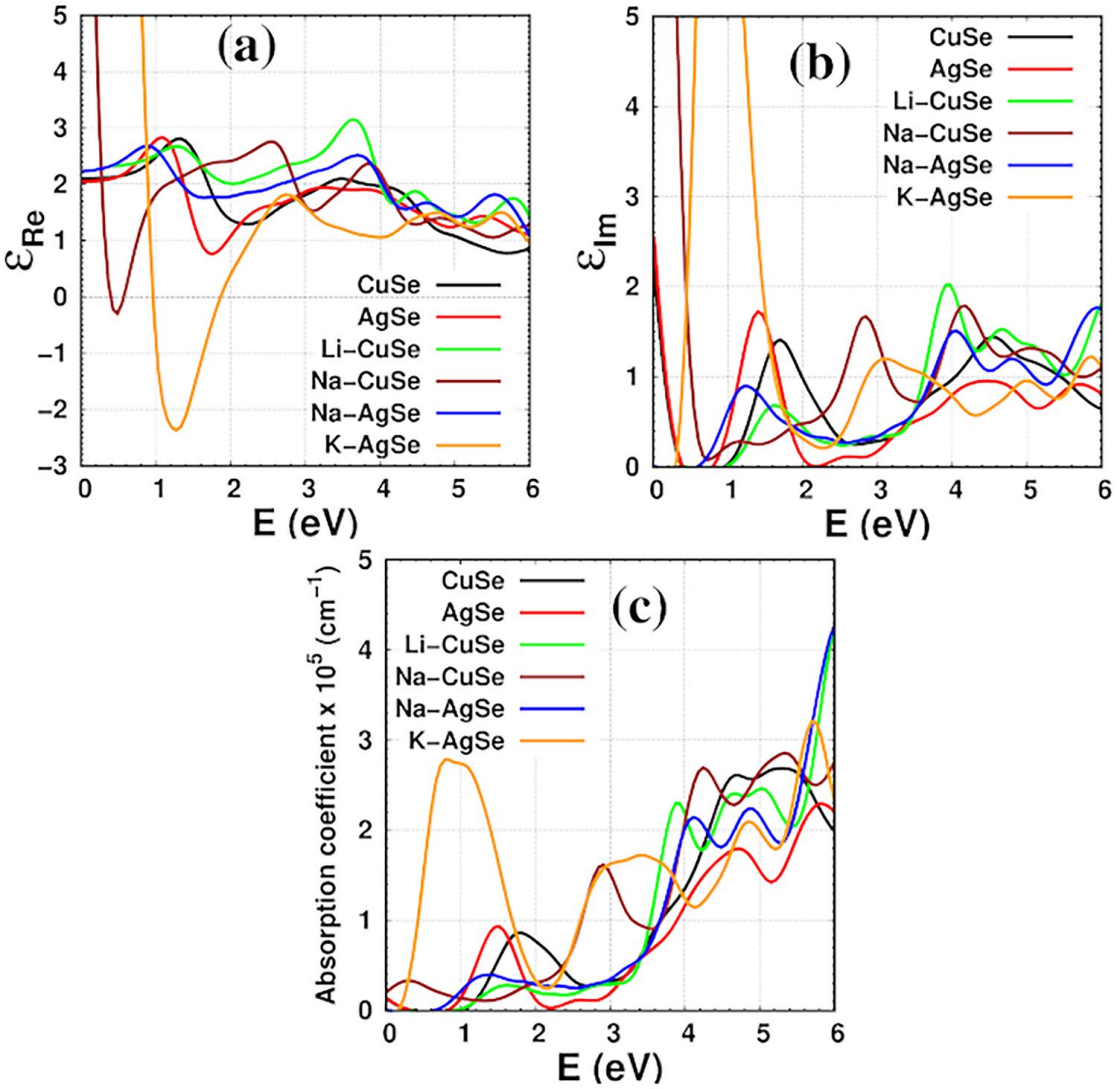
(**a**) Real part of the dielectric function, (**b**) imaginary part of the dielectric function, and (**c**) the absorption coefficients for pristine and functionalized CuSe and AgSe monolayers.

Figure 5a demonstrates the static dielectric is 2.1 for CuSe and AgSe monolayers, 2.3 for the Na-AgSe and Li-CuSe systems, 10 for the Na-CuSe, and 5.9 for the K-AgSe system where the static dielectric is the *ε*_Re_(*E* = 0). There are distinct peaks in the energy range of 1.0–1.4.0 eV for investigated layers, except for Na-CuSe and K-AgSe systems. The position of these distinct peaks depends on the structure. Na-CuSe and K-AgSe systems have the lowest minimum at 0.4 eV and 1.4 eV, respectively. In the energy range from 1.6 to 2.6 eV, the real dielectric part of Li-CuSe, Na-CuSe and Na-AgSe is larger than the real dielectric part of the corresponding pristine monolayers (CuSe and AgSe). For the imaginary part of the dielectric function, Fig. 5b, the onset point of the spectra is related to the kind of structure (metal or semiconductor). For metal systems (CuSe, AgSe and Na-CuSe), there is a value for the imaginary dielectric parts at *E* = 0. However, the onset point is related to their bandgaps for semiconducting monolayers (Li-CuSe, Na-AgSe, K-AgSe). Notice that the first peak appears are at 1.6 eV (K-AgSe), 1.1 eV (Na-AgSe), 1.4 eV (AgSe), 1.6 eV (Li-CuSe) and 1.8 eV (CuSe). Furthermore, there is another distinct peak at 2.8 eV for Na-AgSe and a wide shoulder from 2.8 to 3.6 eV for K-AgSe. Above 4.0 eV, at least, there is a peak for these structures. The imaginary portions of the dielectric function are more closely associated with the absorption spectra. The absorption curves’ onset point and peak position, Fig. 5c, are the same as in the corresponding imaginary components. The K-AgSe monolayer is the best monolayer to absorb infrared radiation (E < 1.6 eV) and visible light (1.6 eV < E < 3.2 eV) as compared to other monolayers. Na-CuSe system can absorb the visible light in the energy range from 2.2 to 3.2 eV. The CuSe monolayer can absorb a portion of visible light in the energy range from 1.8 to 2.4 eV better than the AgSe monolayer.

## Conclusion

Using first-principles calculations, we thoroughly study the atomic structure, electrical and optical properties of AgSe and CuSe monolayers, and the effects of Li, Na, and K decorating. The CuSe and AgSe monolayers both show metallic properties at the ground state. The functionalized Li(Na)-CuSe and Na(K)-AgSe systems are dynamically stable when functionalized with Li, Na, and K atoms to the CuSe and AgSe monolayers. On the other hand, the K-CuSe and Li-CuSe structures are dynamically unstable. It is interesting to note that, the functionalization of Li and Na (K and Na) atom into the CuSe (AgSe) systems can open the band gaps, resulting in the actualization of metal to semiconductor transitions. By varying the adsorption heights, the Na-CuSe/AgSe system properties may be controlled, leading to a shift in band gaps and the transition from metal to semiconductor. Additionally, we find that the best candidate monolayer to absorb visible light and infrared radiation is the K-AgSe system. As a result, our research clarifies the ornamentation of these 2D material. It may encourage further research into their production for use in present-day nanodevices and future uses.

### Supplementary Information

Supplementary Information.

## Data Availability

All data generated or analyzed during this study are included in this published article and its supplementary information files.
