# Supplementary material for: Surface modification of XSe (X = Cu and Ag) monolayers by grope 1 elements: A metal to semiconductor transition by a first-principles perspective
Source: Sci Rep. 2024 Jun 3;14:12695. doi: 10.1038/s41598-024-63580-0 (PMC11148093; doi:10.1038/s41598-024-63580-0)
Supplement: Supplementary file 1 — Supplementary Information. [file 41598_2024_63580_MOESM1_ESM.docx]

Supplementary Information (SI)

Surface modification of XSe (X=Cu and Ag) monolayers by grope 1 elements: A metal to semiconductor transition by a first-principles perspective

A. Bafekry^1,2^ ^∗^, M. Faraji^3^, S. Hasan Khan^4^, M. M. Fadlallah^5^, H. R. Jappor^6^, B. Shokri^2,7^,

M. Ghergherehchi^8,^ ^†^, and Gap Soo Chang^9^

^1^Department of Physics, University of Guilan, 41335-1914, Rasht, Iran

^2^Department of Physics, Shahid Beheshti University, 19839-63113, Tehran, Iran

^3^Micro and Nanotechnology Graduate Program, TOBB University of Economics and Technology, Sogutozu Caddesi No 43 Sogutozu, 06560, Ankara, Turkey.

^4^Department of Electrical and Electronic Engineering (EEE), Khulna University of Engineering & Technology (KUET), Khulna-9203, Bangladesh.

^5^Department of Physics, Faculty of Science, Benha University, 13518 Benha, Egypt

^6^Department of Physics, College of Education for Pure Sciences, University of Babylon, Hilla, Iraq

^7^Physics Department and Laser-Plasma Research Institute Shahid Beheshti university Evin,19839, Tehran, Iran

^8^Department of Electrical and Computer Engineering, Sungkyunkwan University, 16419 Suwon, Korea

^9^Department of Physics and Engineering Physics, University of Saskatchewan, Saskatoon, SK S7N5E2, Canada

∗ email address: [**bafekry.asad@gmail.com**](mailto:bafekry.asad@gmail.com)


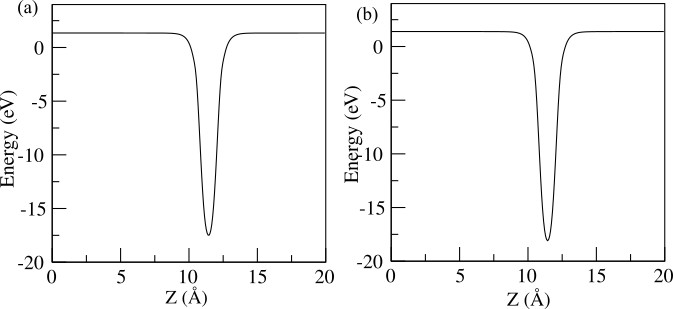


Fig. S1. Electrostatic potential of functionalized (a) CuSe and (b) AgSe monolayers.


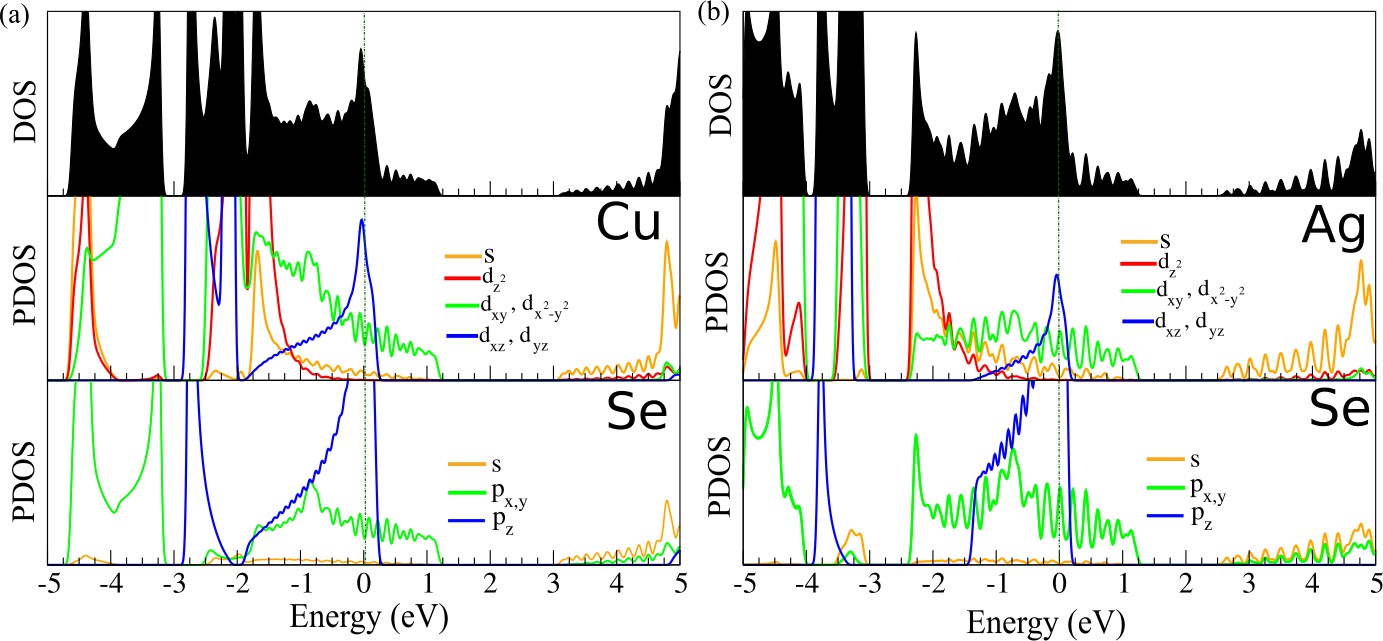


Fig. S1. Orbital projections of the (a) CuSe and (b) AgSe monolayers. The Fermi energy is aligned to 0 eV and shown by a vertical dash-dotted green line.

# S1


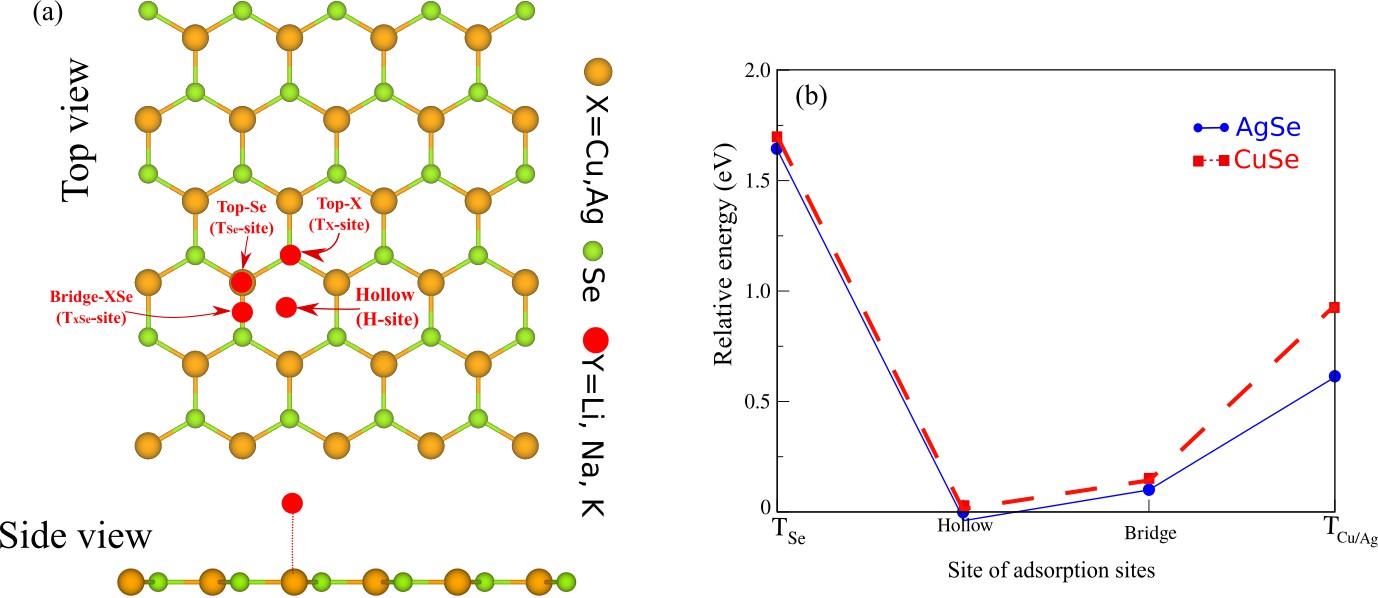


Fig. S3. (a) Schematic view of possible adsorption sites of adatom (Li, Na, K) on the XSe (X=Cu,Ag) monolayer. (b) Relative total energies for sites of Li adsorption on the CuSe and AgSe monolayers.

S2


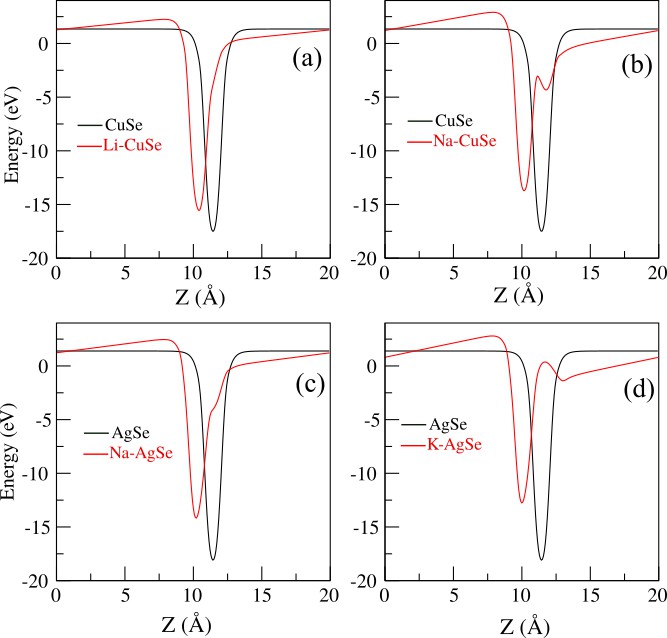
Fig. S4. Electrostatic potential of functionalized (a) Li-CuSe, (b) Na-CuSe, (c) Na-AgSe,

(d) K-AgSe monolayers. Electrostatic potential of pristine monolayers is shwon by black line.

S2
